# Host–parasite biology in the real world: the field voles of Kielder

**DOI:** 10.1017/S0031182014000171

**Published:** 2014-03-10

**Authors:** A. K. TURNER, P. M. BELDOMENICO, K. BOWN, S. J. BURTHE, J. A. JACKSON, X. LAMBIN, M. BEGON

**Affiliations:** 1 Institute of Integrative Biology, University of Liverpool, UK; 2 National Centre for Zoonosis Research, University of Liverpool, UK; 3 Laboratorio de Ecología de Enfermedades, Instituto de Ciencias Veterinarias del Litoral, Universidad Nacional del Litoral – Consejo de Investigaciones Científicas y Técnicas (UNL – CONICET), Esperanza, Argentina; 4 School of Environment & Life Sciences, University of Salford, UK; 5 Centre for Ecology & Hydrology, Natural Environmental Research Council, Edinburgh, UK; 6 Institute of Biological, Environmental and Rural Sciences, University of Aberystwyth, UK; 7 School of Biological Sciences, University of Aberdeen, UK

**Keywords:** field vole, *Microtus agrestis*, Kielder, host, parasite, infectious disease, dynamics

## Abstract

Research on the interactions between the field voles (*Microtus agrestis*) of Kielder Forest and their natural parasites dates back to the 1930s. These early studies were primarily concerned with understanding how parasites shape the characteristic cyclic population dynamics of their hosts. However, since the early 2000s, research on the Kielder field voles has expanded considerably and the system has now been utilized for the study of host–parasite biology across many levels, including genetics, evolutionary ecology, immunology and epidemiology. The Kielder field voles therefore represent one of the most intensely and broadly studied natural host–parasite systems, bridging theoretical and empirical approaches to better understand the biology of infectious disease in the real world. This article synthesizes the body of work published on this system and summarizes some important insights and general messages provided by the integrated and multidisciplinary study of host–parasite interactions in the natural environment.

## INTRODUCTION

Almost 90 years ago, Charles Elton drew attention to the potential importance of parasites in the dynamics of natural populations (Elton, 1924; Elton *et al.* 1931). Nonetheless, for many decades in the 20th century, both this and the dynamics of the parasites themselves were Cinderella subjects in ecology, neglected in comparison with their sister interactions, predation and competition. The tide was turning, though, in the 1970s and early 80s, and may be said to have done so decisively with the publication of seminal papers by Anderson and May at that time (Anderson and May, 1979; May and Anderson, 1979). However, these papers and many that followed were theory-oriented, and field data with which to confront these theories were then – and remain still – in relatively short supply.

**Fig. 1.**
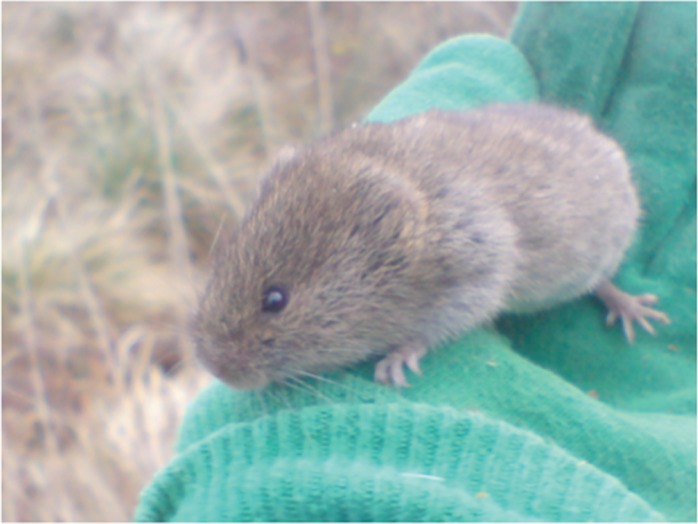
The field vole, *Microtus agrestis*.

In Liverpool in the 1990s, separate lines of research were being pursued on host–parasite dynamics in laboratory populations of moths (Begon *et al.* 1996) and the distribution of a zoonotic pathogen, cowpox virus, in natural populations of rodents (Crouch *et al.* 1995). There were obvious attractions in moving from the laboratory to the field, in looking more deeply into the ecology of cowpox virus, and in re-visiting host–parasite dynamics in rodents, the favoured hosts of Charles Elton (himself a Liverpudlian). Work was initially on bank voles (*Myodes glareolus*) and wood mice (*Apodemus sylvaticus*) in woodland habitats on the Wirral peninsula, near Liverpool, where both species exhibited clear annual (autumn) peaks in abundance, but no demonstrable multi-annual patterns and indeed only moderate variation in abundance from year to year (Hazel *et al.* 2000). Then, from the early 2000s, the focus began to shift to populations of field voles (*Microtus agrestis*) (Fig. 1) in Kielder Forest on the England–Scotland border, living in grassland habitats and exhibiting multi-annual cycles in their abundance (Lambin *et al.* 2000). These latter are the focus of this review. However, where related work on bank voles and wood mice sheds light on the Kielder Forest field vole system, this too is described.

Studying hosts with cyclic dynamics offers two particular advantages in the context of host–parasite dynamics in natural populations. First, where the aim is to investigate the role of parasites in driving host dynamics, it is necessary to know what is ‘signal’ in those dynamics (and hence liable to explanation) and what is simply noise. In cyclic systems there is a clear dominance of signal over noise. Second, many key aspects of the dynamics of the parasites themselves, including those concerned with transmission and host condition, are dependent on host density. In order to have the statistical power to study these effects in a natural population, a system must provide observations across a whole spectrum of densities. Systems with cyclic dynamics are likely to do this and to do so in a predictable way that allows scientific investigation to be planned, as highlighted by the extensive research performed on another cyclic system, the Soay sheep of St Kilda (Clutton-Brock and Pemberton, 2004).

## BACKGROUND TO THE SYSTEM

The Kielder Forest area has a long history of being affected by irruptions of field voles, sometimes of plague proportions (Elton *et al.* 1935; Elton, 1942). Indeed, some of the first steps in studying the population ecology of disease by researchers in Charles Elton's Bureau of Animal Populations were made on field voles in or near Kielder Forest (Chitty, 1954, 1996). The Liverpool group's move from studying local bank voles and wood mice populations to work on the field voles of Kielder was greatly facilitated by the initiation of a collaboration with Xavier Lambin's group at the University of Aberdeen. Prior to the collaboration, the Aberdeen group had been investigating the processes behind temporal and spatial dynamics of field voles (e.g. Mackinnon *et al.* 2001), including the role of predators in shaping spatial dynamics (Petty *et al.* 2000; Sherratt, 2001), and had been utilizing both monitoring and field-experimental approaches (Ergon *et al.* 2001*a*, *b*; Graham and Lambin, 2002). In addition, a study of raptor dynamics has entailed monitoring field vole abundance since 1983 (Petty, 1992) and thus the spatiotemporal dynamics of field voles in Kielder have been exceptionally well characterized. Data reveal a pattern of multi-annual fluctuation with peak vole densities occurring at a 3–4 year interval, followed by steep population collapses usually taking place in summer during the vole breeding season, and followed by up to a year with little noticeable population growth (Lambin *et al.* 2000). The ‘Chitty effect’ (Chitty, 1952; Boonstra and Krebs, 1979), whereby adults in the high-density phase of the population cycle exhibit larger average body mass than during the low phase, is apparent in the Kielder field voles, seemingly through prolonged growth periods rather than increased growth rates (Burthe *et al.* 2010). Within a single grass patch, maximum densities span 5–770 voles ha^−1^ (Burthe *et al.* 2006), but at a landscape scale the span is 50–220 voles ha^−1^ (Lambin *et al.* 1998, 2000; Mackinnon *et al.* 2001). Critically, there is no evidence that voles ever go extinct at any spatial scale in the system. This has implications for the dynamics of species linked to voles, as neither predators nor pathogens are expected to experience extinction–recolonization dynamics in the system.

The population cycles are generally asynchronous among populations across Kielder, although populations situated close together often fluctuate in a synchronous manner (Lambin *et al.* 1998). Indeed, vole spatial dynamics in Kielder Forest were, at least for a time, spatially organized in travelling waves (Lambin *et al.* 1998; Bierman *et al.* 2006). These spatial dynamics, in addition to their intrinsic interest, provide scope for substituting space for time in sampling host populations at a range of densities.

The field voles have ‘fast’ life histories typical of microtine rodents, with high fecundity (an average litter size of five), a low age at maturation for some seasonal cohorts (as little as 28 days old for spring- and early summer-born females), and birth intervals by members of overlapping cohorts as short as 21 days during a breeding season. The field vole breeding season, for the most part, coincides with the plant growing season. However, for individuals born in the second half of the breeding season, reproduction is typically delayed until the next spring. There is therefore a strong seasonality to reproduction and the production of cohorts of susceptible individuals. Juveniles and subadults, but also females breeding in the year of birth, have non-defended home ranges, though overwintered females do tend to defend territories (Pusenius and Viitala, 1993). Dispersal is primarily by subadults. Thus there is also a distinct seasonal pattern to the spatial range and the number of individuals with whom individuals make potentially infectious contacts.

Kielder Forest is intensely managed for timber production. Hence, grassland areas are restricted to roads, unplanted river margins and restock sites where dense grass cover establishes 2–3 years after rotational timber harvesting and persists for 10–12 years after planting. Restock sites are typically 5–25 ha in size and are embedded in a matrix of dense spruce plantation with no grass cover. The field vole is a grass-eating species that therefore relies more than bank voles on well-vegetated areas. The bank vole, common shrew (*Sorex araneus*) and pygmy shrew (*Sorex minutus*) share many arthropod disease vectors and some pathogens with field voles and also mostly occupy grassy areas, whereas wood mice use both the forested landscape matrix and the semi-isolated grassland patches.

## THE DATASETS

**Fig. 2.**
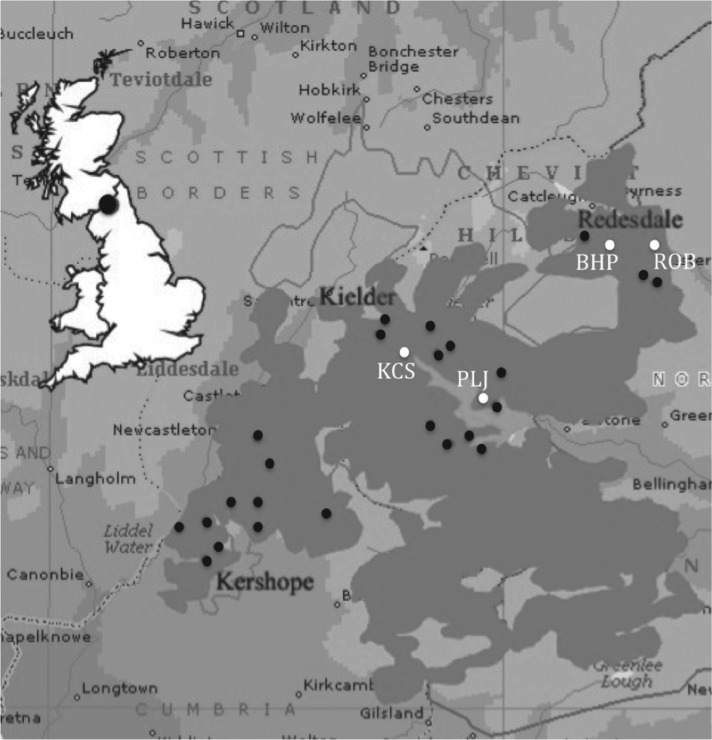
Map of longitudinal (white, labelled) and cross-sectional (all) sites within Kielder Forest and surrounding area.

Many of the Kielder studies used all or part of two datasets, and so the nature and derivation of these are described first in outline. The one used most often was a longitudinal, time series dataset (for full details see, for example, Burthe *et al.* 2006). Field voles were trapped in four similar-sized clear-cuts, in two areas of the forest approximately 12 km apart, between May 2001 and March 2007 (Fig. 2). Two sites in the Kielder catchment, Kielder Site (KCS) and Plashett's Jetty (PLJ) are situated 4 km apart. Two further sites in the adjacent Redesdale Forest catchment, Black Blake Hope (BHP) and Rob's Wood (ROB), are 3·5 km apart. These four populations are far enough apart, with sufficient forest between them, to be considered as effectively independent replicates. Populations were trapped in ‘primary’ sessions every ∼28 days from March to November, and every ∼56 days from November to March. Each site had a permanent 0·3 ha live-trapping grid consisting of 100 Ugglan Special Mousetraps (Grahnab, Marieholm, Sweden), in optimal habitat dominated by *Deschampsia caespitosa, Agrostis tenuis* and *Juncus effusus* grasses. Traps were set at 5 m intervals and baited with oats/wheat and carrots. Traps were pre-baited 3 days before each trapping session, set at approximately 18:00 on the first day and checked five times (‘secondary sessions’) at roughly 12 h intervals, starting and ending at dawn and dusk, respectively. Mass, sex, body condition and reproductive status (assigned according to the external appearance of reproductive organs) was recorded at the time of first capture in each primary session. Individual animals were identified using subcutaneous microchip transponders (AVID plc, East Sussex, UK) injected into the skin at the back of the neck. Total population size was estimated by capture–recapture methods assuming a closed population, from data within a primary session.

A 20–30 *μ*L blood sample was taken from the tail tip of each individual each primary session, usually in the first secondary session in which it was caught. These provided the material for pathogen diagnoses and haematological measurements (see later sections). The presence and (in some cases) the number and identity of ectoparasites were also noted. In addition, the presence of external skin lesions characteristic of late-stage vole tuberculosis (caused by the bacterium *Mycobacterium microti*, see below) was noted in the field. Hence, in those cases where individuals were recaptured in one or more primary sessions, the time course of infections in individuals and transitions in individual status could be monitored, as well as the profiles of whole populations being followed over time.

The second, cross-sectional dataset was derived from traps set bi-annually in March (spring) and September (autumn) in 27 grass-dominated clear-cut sites (5–12 ha) within the three adjacent catchments of Kielder Forest (for full details see, for example, Telfer *et al.* 2007*a*). This design used ‘destructive’ sampling methods, in which captured voles were euthanized. There were 12 sites located in the Kielder catchment, 10 within the Kershope catchment and 5 sites within the Redesdale catchment (Fig. 2). The minimum and maximum inter-site distances were 0·4 and 36·9 km respectively. Within each clear-cut, small mammals were sampled using the small quadrant design (Myllymaki *et al.* 1971): a 15×15 m trapping square was established in good quality field vole habitat and three Ugglan traps were set at each corner. Other procedures were as described for the longitudinal study.

An inherent disadvantage of cross-sectional destructive sampling is that it provides only a snapshot of each host within the dynamics of a host–parasite system; one cannot distinguish causation from mere association, because all measurements are coincident. Nonetheless, a major advantage of such methods is that additional data can be obtained from euthanized individuals (e.g. larger blood volume, organ samples, confirmed reproductive status, etc.).

The dataset used for the immunological work, and also providing data for genetic studies (see later sections), was separate from these (Jackson *et al.* 2011). It came from repeated trapping at two spatially separate sites from February 2008 to March 2009 and a further two from April 2009 to March 2010, and had both longitudinal and cross-sectional components. Each site contained a live-trapping grid (∼0·375 ha) of 150 (10×15) regularly spaced traps (3–5 m intervals) placed in optimal habitat for the longitudinal study. There were also satellite transects on each (with traps spaced at ⩾5 m intervals) from which 10 animals per month per site were sampled destructively (to allow a wider range of immunological measurements) for the cross-sectional component of the study. At each site, there were monthly trapping sessions from February/April to November, during which capture–recapture and destructive samples were taken. Then, in November and again in the following March, larger numbers of animals were destructively sampled both from the transects and from the grid habitats. Other procedures were as described previously.

## EPIDEMIOLOGY OF INDIVIDUAL PATHOGENS

The Kielder field voles are subject to infection by a number of endemic micro- and macroparasite species, of which the microparasite community has been the most extensively studied (Table 1). Many of these species are zoonotic or related to pathogens of medical or veterinary importance and include directly transmitted and vector-borne pathogens, and the ectoparasitic vectors themselves. Here, we provide brief summaries of the background biology and epidemiological patterns displayed by the pathogen species most closely studied in Kielder, followed by more thematic sections drawing together results from ranges of pathogens.

**Table 1.** Summary of microparasites studied in Kielder field voles. Adapted and expanded from Telfer *et al.* (2010)

Species	Type	Transmission mode	Primary site of infection	Infection length	Clinical signs/effects on fitness
Cowpox virus	Virus	Direct	Respiratory tract and lymphoid tissues (monocytes and macrophages)	Self-limiting (4 weeks)	No apparent clinical signs, but reductions in fecundity and survival
*Mycobacterium microti* (vole tuberculosis)	Bacterium	Direct	Unknown but cutaneous, respiratory tract and lymphoid tissues likely	Unknown but most likely chronic (lifelong)	Characteristic skin lesions
*Anaplasma phagocytophilum*	Bacterium	Vector-borne (ticks)	Granulocytes	Self-limiting (4–8 weeks)	Transient cytopenias
*Bartonella* spp.	Bacterium	Vector-borne (fleas)	Erythrocytes	Self-limiting (4–8 weeks)	No apparent clinical signs
*Babesia microti*	Protozoan	Vector-borne (ticks)	Erythrocytes	Chronic (lifelong)	Haemolytic anaemia, generally subclinical
*Trypanosoma* (*Herpestoma*) *microti*	Protozoan	Vector-borne (fleas)	Blood	Unknown, probably self-limiting (a few weeks)	Anaemia

### Cowpox virus

Cowpox is an orthopoxvirus endemic throughout European and western Asian rodent populations (Baxby and Bennett, 1999). Despite its name, cowpox virus rarely infects cattle and is actually most often diagnosed in domestic cats (Cavanagh *et al.* 2004). The virus is also a zoonosis, although human cases are rare (Baxby *et al.* 1994). Field voles, bank voles and wood mice appear to be the primary reservoir hosts in the UK (Chantrey *et al.* 1999), with Kielder field voles exhibiting a prevalence of 28–100%, peaking in mid to late summer (Burthe *et al.* 2006). A summer peak in seropositivity was also observed in the Liverpool populations of bank voles and wood mice, though at lower prevalences (Hazel *et al.* 2000). Animals infected with the virus develop an antibody response after around 2 weeks and remain infected for approximately 4 weeks (Chantrey *et al.* 1999).

Further analysis showed that both the number infected with cowpox virus and the prevalence of infection increased with total population size (Begon *et al.* 2009*a*). However, whereas previous work in bank voles had suggested a threshold abundance, below which infection was not found (Begon *et al.* 2003), evidence for such a threshold in field voles was at best equivocal, in spite of the wide range of abundances sampled (Begon *et al.* 2009*a*).

Field vole abundance was most strongly correlated with contemporary values of numbers infected and prevalence. However, in the case of the numbers of susceptible hosts, the strongest correlations were with values 1–2 months preceding the values for numbers infected and prevalence. Thus, in transfer function analyses (a statistical technique aimed at identifying drivers of an output – in this case, new infections – in terms of potential inputs), as epidemiological theory would predict, the number of ‘susceptibles’ (which ‘drive’ new infections) were much more effective than those of total vole abundance (of which susceptibles are only a component) in predicting future numbers infected. Nonetheless, while monitoring the number of susceptible individuals has most to offer, the results suggest that monitoring overall abundance, which is much more common and more easily achieved, may nonetheless provide valuable insights into the dynamics of infection (Begon *et al.* 2009*a*).

The seasonality of cowpox virus dynamics was examined further by Begon *et al.* (2009*b*). The timing of seasonal peaks in new infection within the year was related to the multi-annual patterns of abundance displayed by the voles, which in turn was associated with both the number and the rate of recruitment of susceptible hosts. A plentiful and sustained supply of susceptible hosts throughout the summer (March–September), such as occurs in the increase phase in the abundance cycle, gave rise to a steady increase in infected hosts and a peak late in the year – often October or November. However, a meagre supply of susceptible hosts more limited in time, such as occurs in a crash year, was often insufficient to sustain an increase in infected hosts, leading to an early peak, around June, followed by a decline. This was in contrast to more predictable seasonal peaks seen in some human infections (Stone *et al.* 2007), the lesson being that to understand seasonal disease dynamics in wildlife populations, the dynamics of the hosts themselves must be fully taken into account.

### Vole tuberculosis

Vole tuberculosis (vole TB) is a chronic, endemic infection of field voles caused by *Mycobacterium microti*, a member of the *M. tuberculosis* complex (van Soolingen *et al.* 1998). Vole TB is a zoonotic infection, having been infrequently recorded in both immunocompromised and immunosuppressed humans (van Soolingen *et al.* 1998; Niemann *et al.* 2000; Emmanuel *et al.* 2007). In voles, TB causes severe clinical pathology in the later stages of the disease, characterized by externally visible cutaneous lesions (Cavanagh *et al.* 2002, 2004; Burthe *et al.* 2008*b*; Kipar *et al.* 2013). The definitive transmission route is unknown. However, wounding has been suggested due to the common occurrence of cutaneous lesions (Burthe *et al.* 2008*b*).

Prevalence of external signs of vole TB showed evidence of delayed density dependence (Cavanagh *et al.* 2004). However, this approach underestimated TB prevalence (Wells, 1946; Cavanagh *et al.* 2004; Burthe *et al.* 2008*b*). Post-mortem examination and culture of infected tissue samples from cross-sectional surveys indicated prevalence over twice that based solely on external signs, with up to 50% of voles infected in some sites (Cavanagh *et al.* 2002; Burthe *et al.* 2008*b*). Prevalence of infection increased with vole mass (a proxy for age) and hence prevalence was highest in spring when the population was mainly comprised of individuals over 6 months of age (Burthe *et al.* 2008*b*).

### *Bartonella* spp

The bartonellae are gram-negative bacteria and facultative intraerythrocytic parasites of a wide range of mammalian species. Transmission mechanisms are not yet fully understood, but arthropods, often fleas, are important vectors (Birtles, 2005; Buffet *et al.* 2013; Morick *et al.* 2013). Several *Bartonella* species are associated with disease in humans or animals.

Up to five species of *Bartonella* circulate concurrently in woodland rodent communities in the UK (Birtles *et al.* 2001; Telfer *et al.* 2007*a*, *b*). Although small mammals have demonstrated a high (40–60%) *Bartonella* prevalence (Birtles *et al.* 1994; Kosoy *et al.* 1997), infections are self-limiting and do not usually result in clinical disease (Telfer *et al.* 2008, 2010). In Kielder, contrasting dynamics of three *Bartonella* species have been recorded, with only *Bartonella grahamii* exhibiting a distinct seasonal pattern and the three species also differing in their likelihood of infecting young or mature hosts (Telfer *et al.* 2007*a*). Interestingly, all species in general exhibited stronger correlations with host dynamics than those of their vectors, supporting the assertion that flea-borne microparasites can often be incorporated effectively into epidemiological models as directly transmitted pathogens (Dye and Williams, 1995).

### Anaplasma phagocytophilum

*Anaplasma phagocytophilum* is a an obligate intracellular bacterial parasite of granulocytes, which was historically associated with causing tick-borne fever in sheep and other livestock (Foggie, 1949; Hudson, 1950). In the 1990s, the zoonotic potential of *A. phagocytophilum* was realized (Chen *et al.* 1994), although different genetic variants appear to have restricted host ranges (Massung *et al.* 2003; Bown *et al.* 2009). Whilst little is known regarding the effects of *A. phagocytophilum* on rodents, it has well-established immunosuppressive effects on livestock (Woldehiwet, 2010). Rodents demonstrate no obvious clinical signs of infection, and longitudinal studies indicate that infection is short-lived, with the majority of individuals testing positive by PCR for only a single month (Bown *et al.* 2003, 2008). Infection prevalence in Kielder field voles may reach 12% in late summer but falls to zero over winter when no nymph or adult ticks are feeding (Bown *et al.* 2009).

Of the two most commonly found ticks in Kielder (see *Ticks*, below), transmission in small mammals appears to be at least predominantly via *Ixodes trianguliceps* rather than *Ixodes ricinus*, as the absence of *I. ricinus* had no significant effect on infection prevalence in field voles (Bown *et al.* 2008). Similarly, infection in both field voles and common shrews follows the seasonal dynamics of *I. trianguliceps* nymphs (Bown *et al.* 2003, 2009, 2011). Further genetic analyses indicated that distinct *A. phagocytophilum* genotypes exist in separate enzootic cycles, possibly as a result of vector specificity (Bown *et al.* 2009).

### Babesia microti

*Babesia microti* is an intraerythrocytic protozoan parasite infecting wild rodents and the major causative agent of human babesiosis in the USA, a potentially fatal tick-borne zoonosis. In common with other members of the *Babesia* genus, *B. microti* requires an ixodid tick vector for the sexual stage of its life cycle. In the UK, this has been identified as *I. trianguliceps* (Randolph, 1991). The great host-specificity of this nest-dwelling tick, which does not readily bite humans, may explain the lack of human *B. microti* infections in Europe. However, the human-biting tick *I. ricinus* is sympatric with *I. trianguliceps* in many areas, including Kielder (see *Ticks*) and may provide a route for transmission to humans.

*Babesia microti* infections in field voles are usually sub-clinical and persistent, with longitudinal studies demonstrating that individuals testing PCR positive remain so for all subsequent captures (Bown *et al.* 2008; Telfer *et al.* 2008). Interestingly, laboratory studies on related bank voles suggest that whilst infections are chronic, sufficient parasitaemia for transmission to ticks to occur is restricted to a window of only 1–4 days post infection (Randolph, 1995). Infection prevalence may reach over 40% in Kielder field vole populations (Bown *et al.* 2008; Telfer *et al.* 2008) and probability of infection has a non-linear relationship with weight, with individuals of 20 g being at highest risk of becoming infected (Telfer *et al.* 2008).

### Trypanosoma (Herpestoma) microti

*Trypanosoma microti* is a stercorarian trypanosome specific to voles (Noyes *et al.* 2002). Trypanosome infections in rodents are generally considered to be of low pathogenicity, but there is some evidence that trypanosomes can cause anaemia in microtine rodents and detrimentally affect female reproduction (Wiger, 1977).

In Kielder, *T. microti* prevalence is highly seasonal, being highest in late summer/autumn and lowest in spring (Smith *et al.* 2005). *Trypanosoma microti* is transmitted by fleas and a positive association between trypanosome prevalence and flea infestation in the previous 1–3 months has been observed in Kielder field voles (Smith *et al.* 2005). Following ingestion of the parasite during a blood meal, it develops in the flea hindgut before being shed in the feces. Infection of a new vole host can then occur via fecal contamination of the skin, or through accidental ingestion of fleas or their feces (Albright and Albright, 1991). However, a study in which flea prevalence was experimentally manipulated demonstrated that vector-independent transmission of *T. microti*, most likely though mechanical transmission as a result of aggressive behaviour, is also of epidemiological significance in Kielder (Smith *et al.* 2006).

### Ticks (Ixodida)

Ticks are amongst the most important arthropod vectors and, as described above, are responsible for *B. microti* and *A. phagocytophilum* transmission among Kielder voles. In the UK, at least five species of tick may feed on rodents (Snow, 1979) of which two, *I. ricinus* and *I. trianguliceps*, are frequently encountered at Kielder (Bown *et al.* 2006, 2008, 2009). Whilst all three stages of *I. trianguliceps* feed upon small mammals (Randolph, 1975), *I. ricinus* is more catholic in its feeding behaviour, feeding on a wide variety of hosts including reptiles, birds and mammals (Arthur, 1963). As such, the exclusion of deer from an area significantly reduced the abundance of *I. ricinus*, but had no detectable effect on *I. trianguliceps* (Bown *et al.* 2008).

Longitudinal studies indicate that the majority of larvae recorded on field voles were *I. ricinus* whilst adult ticks were almost exclusively *I. trianguliceps* (Bown *et al.* 2009). Seasonal fluctuations in the abundance of ticks feeding on voles were apparent, with peaks of *I. ricinus* larvae in late spring/early summer, whilst *I. trianguliceps* larvae peak abundance occurs in late autumn (Bown *et al.* 2009). Nymph and adult ticks were recorded in much lower numbers with no obvious peak, but were largely absent between November and April (Bown *et al.* 2009). Male voles were more likely to be infested with nymphal or adult ticks, and mature males were more likely to be infested with larvae of either tick species (Bown *et al.* 2008). The presence of larvae increased the probability of nymphs or adults on a vole and vice versa (Bown *et al.* 2008).

### Fleas (Siphonaptera)

A number of rodent-specific and generalist flea species are known to inhabit Kielder Forest, including *Peromyscopsylla spectabilis, Ctenophthalmus nobilis vulgaris, Megabothris walkeri, Malaraeus penicilliger, Rhadinopsylla pentacanthi* and the largest British species, the mole flea (*Hystrichopsylla talpae talpae*) (Smith *et al.* 2005; Jackson *et al.* 2011; Turner *et al.* 2011). Fleas which commonly infest field voles exhibit seasonal dynamics and are known to peak in mid-late summer (Smith *et al.* 2005). Within Kielder, these species have thus far primarily been studied in the context of their transmission of *Bartonella* spp. and *T. microti* (see preceding sections). However, Telfer *et al.* (2007*a*) demonstrated that the probability of flea infestation is density- and delayed density-dependent; voles from clear-cut sites with high densities the preceding autumn were more likely to be infested. Conversely, field voles were less likely to be infested if found in a currently or recently high-density population, suggesting an intra-specific dilution effect whereby the flea population is divided among a greater number of hosts.

### Others

Many other endemic pathogens and parasites of the Kielder field voles are known but have not yet been studied in great detail (see Table 2 for a summary of known macroparasites). However, genetic associations with resistance to nematodes and cestodes have been examined (see *Genetics* section). Research into the impact of these less well-studied pathogens is on-going, and there will undoubtedly be currently undiagnosed pathogens circulating within the field vole populations, particularly microparasites, which will warrant further study.

**Table 2.** Macroparasite species observed in Kielder Forest field voles

Nematodes	*Syphacia nigeriana*
	*Trichuris arvicolae*
	*Heligmosomoides laevis*
Cestodes	*Taenia polyacantha*
	*Taenia taeniaeformis*
	*Taenia mustelae*
	*Anoplocephaloides dentata* aff.
	*Paranoplocephala* sp.
	*Rodentolepis* (=*Hymenolepsis*) *asymmetrica*
	*Arostrilepis horrida*
Mites	Laelapidae
	Listrophoridae
	Myobiidae
	Ear mites
Lice	*Hoplopluera acanthopus*

## TRANSMISSION DYNAMICS

Our work on transmission dynamics, largely focused on cowpox virus and aimed at testing widely held theoretical assumptions using empirical data, began on the Liverpool time series. We initially performed a rather unsophisticated analysis of numbers of infected and susceptible hosts to examine the transmission dynamics of cowpox virus (Begon *et al.* 1998, 1999). We examined dynamics within both wood mice and bank voles to ask, first, whether the density-dependent mode of transmission conventionally assumed – especially in modelling studies – for directly but not sexually transmitted infections, was in fact appropriate (as opposed, for example, to frequency-dependence, where the contact rate between hosts is assumed to remain constant irrespective of density). We also compared transmission rates within and between species. This is important for two reasons: first, for the insights it provides on whether coexisting wildlife hosts should be considered joint or independent reservoirs of infection, and hence whether interspecific dilution or amplification effects are possible (Begon, 2008). Second, it allows an assessment to be made from field data of the strength of ‘apparent competition’ in a host–host–pathogen system, whereas previously this has largely been the subject of theoretical analysis (e.g. Begon and Bowers, 1994). Aspects of the same questions were also examined by the analysis of spatiotemporal cowpox data to assess, first within species, the spatial and temporal scales over which an infectious individual poses a risk of infection to a susceptible one (Carslake *et al.* 2005). The same technique was then applied between species (Carslake *et al.* 2006), in both cases asking, in essence, ‘who acquires infection from whom?’.

The results called into serious question the assumption that susceptible and infectious hosts mix at random and hence that transmission of cowpox virus is ‘density-dependent’. Our time series analysis, for each species in isolation, indicated that frequency-dependent transmission (conventionally assumed to apply to sexually transmitted diseases) was superior to density-dependent transmission as a descriptor of the dynamics (Begon *et al.* 1998). A *K*-function analysis confirmed that an infectious individual posed a measurable risk of infection for a period roughly equal to the infectious period itself, about 4 weeks. It also indicated that this risk was detectable only at spatial scales within the species’ known home ranges (Carslake *et al.* 2005). These results therefore suggest a rather general conclusion, namely that random mixing may have been too readily assumed, and that many diseases that are not sexually transmitted may nonetheless be socially transmitted, with essentially the same transmission dynamics.

Moreover, the time series analysis of the two species together indicated that between-species transmission was rare, in spite of the species occupying not only the same general habitat but often even sharing burrows (Begon *et al.* 1999). The *K*-function analysis confirmed this (Carslake *et al.* 2006). Thus, for cowpox virus at least, bank voles and wood mice act as effectively independent reservoirs. Similarly, while the potential for apparent competition between bank voles and wood mice mediated by cowpox virus undoubtedly exists, since the virus depresses the birth rate and possibly the survival of both host species (see *Fitness effects* section), it is likely to be insignificant in practice because the pathogen is so rarely transmitted from one species to the other.

A further study connected to these was carried out by Telfer *et al.* (2005*a*) examining the interaction between bank voles and wood mice and two of their shared pathogens, *Bartonella birtlesii* and *Bartonella taylorii*, in Ireland. The prevalence of both, which occur only in wood mice in Ireland, declined significantly with bank vole density. Results were therefore consistent with there being a dilution effect (Norman *et al.* 1999), a phenomenon which despite its high profile and the recent controversy it has attracted (e.g. Randolph and Dobson, 2012) is still short of good case studies (see *Fleas* section for an example of another possible dilution effect in Kielder).

Although the Kielder time series could not further our understanding of between-species dynamics, focusing as it did on a single species, it was possible to use the field vole data to examine much more thoroughly the question of the nature of the transmission function itself (Smith *et al.* 2009). Rather than simply comparing density- and frequency-dependent transmission, the analysis asked where on the spectrum between density- and frequency-dependence the true function might be, and also whether that functional form, or indeed the strength of transmission itself, might vary seasonally. In fact, results showed that, overall, transmission of cowpox virus amongst field voles was neither frequency- nor density-dependent. On a scale encompassing 0 (density-dependence) and 1 (frequency-dependence), the observed value of the scaling exponent relating contact rate to density was 0·62. This was significantly different from both 0 and 1 (credibility interval 0·49–0·74), appropriate for a transmission function that increases linearly with host density at lower densities (density-dependence) but tends to saturate as density increases further (approaching frequency-dependence).

Furthermore, when models were examined that allowed parameters to vary seasonally, it appeared, first, that transmission was more readily achieved in winter, perhaps because susceptibility to infection is greatest then. Secondly, the overall picture of transmission lying between density- and frequency-dependence seemed to be hiding a pattern in which transmission was closer to density-dependence in the winter and closer to frequency-dependence in the summer. This is plausible insofar as field voles defend territories much more actively in the breeding season (summer), such that contact will be with neighbours and hence relatively independent of overall density. In winter, mixing is not so constrained, hence contact rates can indeed be expected to increase with density. Repeatedly, therefore, these transmission studies, whether within or between species, have emphasized that once data are collected from natural populations, conventional, widely held assumptions may be found wanting.

## FITNESS EFFECTS OF INFECTION

The impact of endemic infections on the fitness of hosts in the wild is poorly understood, with studies tending to be cross-sectional or to focus on epidemic or emerging infections. Changes in host population dynamics may arise through impacts on host survival and/or fecundity rates. Longitudinal, experimental and modelling work investigating the prevalence of a suite of pathogens in field voles at Kielder has greatly advanced our understanding of the impacts of endemic infections, indicating that negative fitness costs can be biologically significant.

Evidence from Kielder suggests that endemic infections negatively impact field vole survival. Individuals infected with cowpox virus had a 22% lower probability of survival than uninfected individuals and, at the population level, survival rates were negatively correlated with cowpox prevalence (Burthe *et al.* 2008*a*). There is also some suggestion that TB has a negative impact on survival (Burthe *et al.* 2008*b*). While not statistically significant, survival of voles following the appearance of an external lesion characteristic of advanced vole tuberculosis tended to be lower than for voles without lesions. As discussed earlier, diagnosis of disease based on lesions underestimates the prevalence of infection and hence the negative impact of late-stage TB would be underestimated due to individuals dying before presenting overt late-stage disease symptoms. A significant decline in body condition of individuals at the time of appearance of the first external lesion further suggests that TB may potentially impact individual fitness. Further effects of infection on host condition are discussed in the next section.

Impacts on host reproduction by pathogens have proven difficult to evaluate due to difficulties in assigning juveniles to parents and measuring reproductive success. However, prevalence of trypanosomes was found to be highest in heavier (older) animals at first capture compared with heavier recaptured animals suggesting that infected animals may be less likely to become territory holders and therefore less likely to breed (Smith *et al.* 2005).

In related work on bank voles and wood mice in the Liverpool populations, cowpox virus infection was both positively and negatively associated with survival, depending on the season; survival rates increased with cowpox prevalence in the summer but decreased during the winter (Telfer *et al.* 2002). This may be related to subtle interactions with effects of cowpox virus on reproduction in these species. Female bank voles and wood mice infected with cowpox virus have been shown to delay maturation, and therefore reproduction, often until the following year (Telfer *et al.* 2005*b*), a response also observed in the laboratory (Feore *et al.* 1997). This delay in reproduction, and the associated energetic costs saved, may be the reason for the increased survival rates for cowpox-infected compared with non-infected individuals in summer.

Modelling work suggests that, theoretically, reduced or delayed fecundity following recovery from infection can influence host population dynamics and induce multi-year cycles if, due to infection, individuals miss the time-window for sexual maturation in their birth year and are thus first able to reproduce the following spring (Smith *et al.* 2008). However, empirical investigation of parameters such as variation in the onset of maturity in infected hosts relative to uninfected hosts would be necessary to support this theoretical prediction.

As discussed in more detail below (see *Co-infection*), the field vole data indicate clearly that infection with one pathogen may frequently imply co-infection with others. The fitness consequences of infection with, say, cowpox virus, may therefore, in practice, be the fitness consequences of infection with cowpox virus and all the other parasites that are consequently more likely to be found in the same host. This sets limits on the relevance of controlled experiments in the animal house on the effects of parasites on host fitness. It also emphasizes that there may often be no clear link between the clinical effects of an individual parasite species and the effects it has on host fitness in statistical analyses carried out at the population level.

## HAEMATOLOGY AND MEASURES OF HOST CONDITION

Variation among individuals and populations in health status and immunocompetence (a host's general ability to resist or tolerate infection) may influence parasite dynamics, as a result of variable susceptibility to infection (see *Immunology* and *Genetics* sections for studies on the immunological and genetic basis of this variation). In human and veterinary medicine the health status of individuals is routinely monitored by measuring selected physiological indices, and haematological parameters are among the indices most extensively used. Nonetheless, the wealth of information they can provide has not always been fully exploited in wildlife populations.

The cellular component of the blood consists of various cell types, each of which has a different function and responds distinctively to infection, stress, nutritional deficit, etc. Although the interpretation of these parameters requires caution, in general, red blood cells (erythrocytes, RBCs) and lymphocytes are important indicators of general health and condition, while the other white blood cells (WBCs) are components of different types of immune responses (Tizard, 2004). Low concentrations of RBCs, caused by blood loss, haemolysis or decreased erythrocyte production, result mainly from deficient nourishment and infection or parasitism (Stockham and Scott, 2002). Lymphocytes, the effectors of acquired immunity, proliferate in response to antigenic stimuli and have a long lifespan in blood, while their numbers decrease (lymphocytopenia) during immunosuppression by glucocorticoids or immunosuppressive infections (Feldman *et al.* 2000; Stockham and Scott, 2002). Therefore, circulating levels of lymphocytes may be useful indicators of current immunological investment. Of the remaining WBCs, blood concentrations of neutrophils increase rapidly as a response to cytokines released during tissue injury and bacterial infection (Tizard, 2004). They are useful proxies for acute inflammatory responses as their levels return to normal soon after antigenic stimulation ceases. Monocytes are found in high concentrations in subacute and chronic inflammatory response caused by bacterial or protozoan infections (Feldman *et al.* 2000; Tizard, 2004).

By evaluating indices of health in wild populations, our knowledge of the dynamics of health and infection may be understood more clearly. Beldomenico *et al.* (2008*b*) investigated haematological dynamics within the Kielder field voles, to determine environmental and host factors associated with indicators of inflammatory response (counts of monocytes and neutrophils) and of condition (lymphocyte and red blood cell counts). Individuals from three field vole populations were sampled monthly for 2 years. Comparisons with individuals kept under controlled conditions facilitated interpretation of field data (Beldomenico *et al.* 2008*b*).

In comparison with humans and domestic animals, which maintain their haematological parameters within constant ‘normal’ ranges while in health, these parameters appeared to be highly variable in wild field voles. This may imply a more widespread distinction between the range of environments experienced by individuals typically found in natural vertebrate populations and those experienced under controlled or cosseted conditions. There was a strong seasonal variation that persisted even after environmental and host factors usually associated with blood cell count variation were considered in the analysis. There were three well-characterized ‘physiological’ seasons. The immunological investment appeared lowest in winter (lowest lymphocyte counts), but red blood cells were at their highest levels and indices of inflammatory response at their lowest, indicating a low infection risk during this period. Spring, when acquisition of breeding territories is expected to lead to acute competition for space and increased aggressive behaviour, was characterized by dramatic changes, with a steep fall in red blood cell counts and peaks in indicators of inflammatory response. During the course of summer–autumn, the parameters gradually returned to their previous levels; red blood cell counts recovered and the indicators of inflammatory response decreased, while the immunological investment increased.

All the haematological parameters were lower in individuals in poor body condition and when preceding population densities were higher. Moreover, the first pregnant females of the year were those with higher values of the haematological metrics, emphasizing the important role of energetics in population dynamics. Indeed, even when RBC counts were ‘high’ in the field, they were lower than in the near-optimal conditions of the animal house (abundant food and low parasitism/infection), suggesting that voles in the natural populations were generally resource and/or energy-limited, and they could therefore only increase their investment in, for instance, neutrophils by a compensatory decrease in their investment in other functions (e.g. the production of RBCs) (Beldomenico *et al.* 2008*b*).

Azurocytes (AZ) are lymphocytes specific to microtine rodents which closely resemble natural killer (NK) cells. They are particularly common in late pregnancy and inducible by progestins both in males and females (Mihok *et al.* 1987; Mihok and Schwartz, 1991). Beldomenico *et al.* (2008*c*) found that the counts of AZ were indeed much higher in pregnant females, and that these counts were positively correlated with past vole density. Males had low prevalences and counts, both for breeding and non-breeding individuals, but they showed a seasonality that varied with age, body condition, and current and past vole density. Also, the occurrence of AZ in males was more likely after they had had low levels of indicators of condition (see following section), suggesting that in males these cells predominantly result from a response to infection.

The strong seasonal variation in health dynamics pinpoints the spring as a period of increased vulnerability, both to infection and other causes of mortality. When population densities have previously been low and body condition is good, female field voles begin reproducing early (Ergon *et al.* 2011). Preceding high densities are followed by a negative impact on all blood cell types, except for AZ in females. Host condition in spring may therefore not only reflect but also determine, in part, whether a year will be in an increase or a decrease phase of the abundance cycle.

## VICIOUS CIRCLES: SYNERGY BETWEEN CONDITION AND INFECTION

The previous section discussed effects of infection on host fitness and condition. Equally, a host's condition may affect its propensity to become and to remain infected. Contact between susceptible hosts and either infectious hosts, vectors or environmental reservoirs is crucial in determining infection risk. However, following exposure to a pathogen, a continuum of outcomes might be seen within a host, ranging from failure of the infection to progress to overwhelmingly high infection intensity. The outcome may depend on characteristics of the pathogen (e.g. strain, infective dose) or of the host (e.g. genotype, condition).

As discussed earlier, the Kielder field voles exhibit characteristic periodic peaks followed by declines, and these dynamics are associated with food shortage and poor condition (Huitu *et al.* 2007). To test the hypothesis that poor host condition increases infection risk, Beldomenico *et al.* (2008*a*) used longitudinal data from replicated wild field vole populations to evaluate whether individuals with reduced indicators of condition were more likely to become infected. Because the community of obligate and facultative parasites to which field voles are exposed is highly diverse, exhaustively testing for all infections is impossible. To overcome this problem, initially generic indices that capture the physiological response to infection were used. Elevated neutrophil counts (neutrophilia) are an indication of acute inflammatory response associated with bacterial infection, and high monocyte counts (monocytosis) are expected in subacute and chronic inflammatory response caused by infections with bacteria or protozoans (Feldman *et al.* 2000). In addition, low peripheral lymphocyte counts (lymphocytopenia, an indication of immunosuppression or poor immunological investment) or low red blood cell (RBC) counts (anaemia, an indication of poor aerobic capacity) were used as haematological indicators of condition (see *Haematology*). The results showed that poor condition increases the probability of infection: individuals with anaemia and lymphocytopenia had increased probabilities of developing monocytosis and higher increments in neutrophils when re-sampled 4 weeks later (Beldomenico *et al.* 2008*a*).

**Fig. 3.**
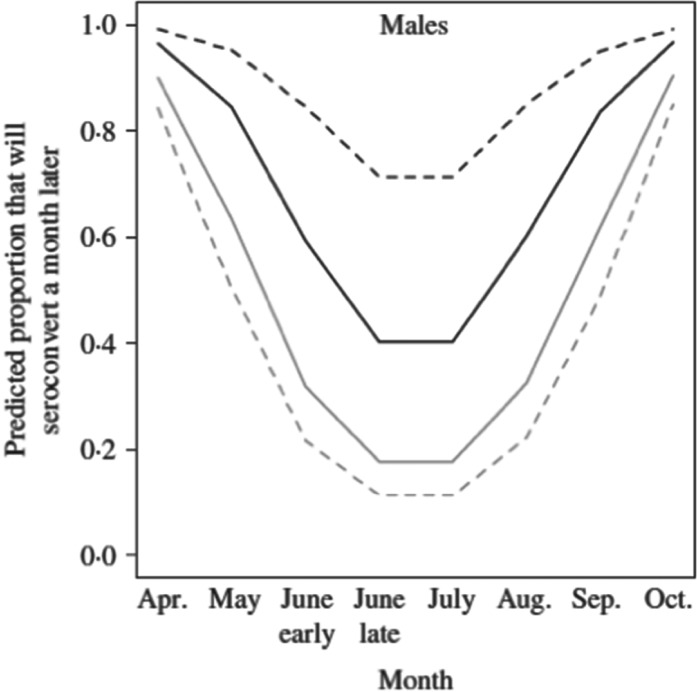
Predicted probability of seroconverting for male field voles from Kielder. Variation by month, body condition score (4 = black lines; 8 = grey lines) and red blood cells (RBCs) (past density fixed at 50). In the simulation, anaemic (dashed lines) represents individuals with 3 million RBCs mL^−1^, and normocytic (solid lines) represents voles with 8 million RBCs mL^−1^. Modified from Beldomenico *et al.* (2009*b*).

The results above provide evidence supporting Lochmiller's hypothesis (Lochmiller, 1996), which states that opportunistic pathogens take advantage of altered host immunocompetence (see *Immunology* section for further discussion on the concept of immunocompetence) during stress periods, consequently regulating wild animal populations. To test this hypothesis in the Kielder system, Beldomenico *et al.* (2009*a*) carried out a nested case-control study that assessed whether susceptible individuals (those who had never had cowpox) with poorer body condition (low degree of fat and muscle cover) had higher probabilities of contracting cowpox over a 4-week period. The results were particularly striking for males. For males caught at the same time, a susceptible individual with poor body condition was twice as likely to contract cowpox as a susceptible male with good body condition; if this individual was also anaemic, the chances were almost quadrupled (Fig. 3). This result supported Lochmiller's hypothesis, and further demonstrated that it holds not only for opportunistic pathogens, but here for an endemic virus.

If this condition-dependent infection risk originates from a reduced resistance of the host, it will not only result in greater propensity to becoming infected of those that are in poorer condition; it may also cause infections of higher intensity, thus resulting in individuals that suffer a more severe disease and are a more significant source of infection. Beldomenico *et al.* (2009*b*) assessed this hypothesis by investigating the temporal relationship between host condition and intensity of infection by the protozoan *Trypanosoma* (*Herpestoma*) *microti* in wild field vole populations. The individuals that developed high levels of parasitaemia were those that had very low lymphocyte counts one month previously.

As noted above, not only can poor host condition predispose individuals to infection; infection itself can have a detrimental effect on condition. Besides their specific pathogenic effects, parasites extract host resources and induce a nutritionally demanding immune response (Sheldon and Verhulst, 1996; Lochmiller and Deerenberg, 2000). There is a clear potential for synergy: poor condition predisposes individuals to infections, which further reduces the condition of the host, which further predisposes the host to infection, and so on. Thus, as previously noted, at the individual level, low haematological indicators of condition precede elevated levels of haematological indicators of infection in wild field voles. However, those individuals with high indicators of infection subsequently experience a decline in their indicators of condition (Beldomenico *et al.* 2008*a*). Furthermore, because individuals in poorer condition are expected to have infections of greater intensity, the resulting deterioration in condition is likely to be even more marked for infections in individuals with a preceding impoverished condition. This was supported by our study on trypanosome dynamics: field voles with decreased indicators of immunological investment developed high intensities of *T. microti* parasitaemia, and subsequently, further declines of these indicators were observed (Beldomenico *et al.* 2009*b*).

The above suggests that small initial differences in host condition caused by resource shortage, competition etc. can become exaggerated and populations might become ‘polarized’ into the weak and the strong (Beldomenico and Begon, 2010). Vicious circles emerge, whereby an individual with an impoverished condition is more prone to developing infections, which are also more likely to be severe; in turn, this results in further deterioration in condition that can eventually and substantially affect its performance and survival. At the population level, a high proportion of individuals in poor condition may cause both a large number of infections and more severe infections, resulting in pathogen exposure dose being greater, with a consequential greater impact on host dynamics (Beldomenico and Begon, 2010). Similar results have been reported in other systems including an observational study on fish (Blanchet *et al.* 2009) and a field-experimental study on mice (Pedersen and Greives, 2008).

These reciprocal effects between host condition and infection might indeed be the mechanism by which parasites exert a control on their host populations, as hosts tend to be more stressed and in poorer condition (thus becoming more vulnerable to their parasites) when their densities are high (Huitu *et al.* 2007; Beldomenico *et al.* 2008*b*).

## CO-INFECTION

While most of the studies at Kielder have focused, as they have in other systems, on a single species of pathogen (and of host), there is no doubt that most hosts, most of the time, are infected by a multiplicity of parasites and pathogens. Questions naturally arise, therefore, regarding the effects of one infection on another. Indeed, some such effects may also occur when infections are consecutive rather than simultaneous. The probability, intensity and length of one infection may be altered by the presence of, or repercussions from, another, as may any effects on host fitness. The idea of ‘vicious circles’ (above) carries within it the implicit acknowledgement that individual infections cannot be considered in isolation.

Experiments in laboratory model systems have demonstrated effects of co-infection on host susceptibility, infection length, and intensity and clinical signs. Studies in wildlife populations and humans, while establishing firmly that positive and negative associations can occur between parasites, have tended to be cross-sectional, with each host providing infection data at one time point only. The time of initial infection is unknown in such studies. There is, therefore, limited scope for determining whether patterns reflect inherent differences between hosts in either susceptibility or exposure to infection, rather than interactions (Telfer *et al.* 2008), or for exploring the impact of infection sequence (Jackson *et al.* 2006). Consequently, in natural populations, the relative importance of interspecific interactions, compared with other factors, in determining the dynamics and structure of parasite communities is only poorly understood.

Telfer *et al.* (2010) used the field vole dataset to examine individual infection risks for a community of microparasites consisting of cowpox virus, *Babesia microti*, the *Bartonella* species taken as a group and *A. phagocytophilum*. Infection risk will depend on both the probability of encountering an infectious dose and the probability of infection given exposure (host susceptibility). The aim was to determine whether susceptibility to infection by one microparasite species was influenced by others. Therefore, for each microparasite, the study investigated whether the other microparasites influenced the probability that a susceptible animal *became* infected at a given time point (*t*_0_). It did so by adding infection status for these other pathogens as explanatory variables to baseline statistical models that accounted for environmental and individual variables (sex, season etc.). This method minimizes the risk of detecting spurious associations, which, in reality, reflect correlated exposure risk (e.g. a positive association simply because both parasites are most prevalent in late summer).

**Fig. 4.**
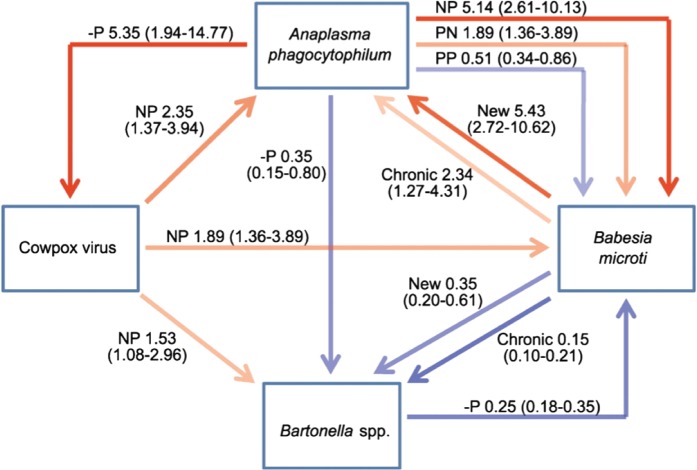
The web of interactions and magnitude of effects between microparasite species in the Kielder field voles. Positive associations (odds ratio [OR]>1) are in red, negative associations (OR<1) in blue, with intensity of line reflecting the magnitude of an effect. 95% confidence intervals of OR shown in parentheses. Infection history associated with effect also noted: N = negative, P = positive. NP therefore signifies no infection at *t*_-1_ and infection at t_0_. Thus, for example, individuals with chronic *B. microti* infections are ∼2 times more likely to be infected with *A. phagocytophilum* (OR = 2·34) while those with new *B. microti* infection are ∼5 times more likely to be infected (OR = 5·43). From Telfer *et al.* (2010).

It was apparent that this community of parasites represents an interconnected web of interactions: effects of other infections on infection risk were both strong and widespread, and connectance within the parasite community was exceptionally high, with evidence detected for all possible pair-wise interactions (Fig. 4). Both positive and negative associations were detected, and their magnitude was frequently considerable: up to 5·5-fold increases in risk and reductions in the odds of becoming infected of the order of 15% compared with uninfected individuals (Fig. 4). Indeed, perhaps most strikingly, in all cases except for cowpox, infection with other parasite species explained more variation in infection risk than factors related to exposure risk and host condition, such as age and season. Moreover, the sizes of the effects of other parasites on infection risk were also similar to, and frequently greater than, other factors. For example, of all the non-infection variables, season generally had the largest effect on infection risk, with seasonal increases in infection probability ranging from approximately 3-fold (*A. phagocytophilum*) to 15-fold (*B. microti*); but these were broadly matched by the magnitude of infection effects (Fig. 4). These results are not explicable by simple co-occurrence of infections in hosts in poor condition since, for a subset of the data, individual variation in indices of host body and haematological condition at the time of infection were explicitly accounted for, and there was no evidence of any reduction in the strength of between-parasite interactions.

Several infections increased susceptibility to other microparasites (Telfer *et al.* 2010). Jackson *et al.* (2009) have shown previously that naturally occurring parasites are capable of exerting immunomodulatory effects on wild rodents, and release from effective control by the immune system is perhaps therefore the most likely explanation, especially when supported by experimental studies. For example, laboratory studies have indicated the importance of immunomodulation for host exploitation by pox viruses (Seet *et al.* 2003), which may explain the positive effect of cowpox virus on susceptibility to other parasites. The same immune-mediated mechanisms might also account for an earlier demonstration that cowpox virus increases the length of *Bar. taylorii* infections (Telfer *et al.* 2008). Thus, mechanisms responsible for increasing susceptibility may also prolong infections in those that do succumb.

Strong reductions in susceptibility caused by other infections were also observed. The largest effect overall was reduced susceptibility to *Bartonella* spp. in individuals infected with *Bab. microti*, and was especially apparent in chronically infected animals, where the odds of infection were 15% of those of uninfected animals (Fig. 4). Resource depletion may play a role here, as both species target erythrocytes (Table 1). Alternatively, negative effects may reflect up-regulation of mediators of a cross-effective Th1 response and therefore could represent an example of immunologically driven ecological interference (see *Immunology* and *Genetics* sections, below).

The study by Telfer *et al.* demonstrates, therefore, that communities of microparasites are structured by strong interactions between species, providing the first evidence from natural populations that such interactions can be driven by effects on susceptibility and have as much impact on infection risk as more commonly considered factors such as host age and season. As field voles are also infected by macroparasites (Table 2), as well as other microparasites, it is likely that the identified relationships represent just one part of an even larger web of interactions. These results also emphasize that the standard practice of classifying individuals in natural populations as infected or uninfected by one parasite alone fails to recognize that much more may be implied by the categorization ‘infected’. For example, as we note above, cowpox virus infection has been associated with major reductions in survival and fecundity. However, in the co-infection study, 39% of those infected with cowpox virus were also infected with *B. microti*, 65% of the remainder had *Bartonella* spp. infections, and overall, 79% were co-infected with at least one of the three microparasites considered. Clearly, even when significant associations between a given infection and host fitness are detected in a wildlife host, attributing the effect to that parasite alone may be unjustified.

A subsequent study applied more sophisticated and novel statistical techniques to the dataset from March 2005–March 2007 and dealt separately with three *Bartonella* species, *Bartonella doshiae, B. grahamii* and *B. taylorii* (Sherlock *et al.* 2013). While broadly confirming previous findings, this new analysis highlighted some possible discrepancies with previous analyses. Once again, *Bab. microti* increased the likelihood of contracting all three *Bartonella* species, whether the *B. microti* infection was acute or chronic. This time, moreover, *B. microti* was also seen to decrease the chances of recovery from all three *Bartonella* infections: that is, *Bartonella* infections were longer when *B. microti* was also present. This had previously also been suggested in the case of *Bar. taylorii* (Telfer *et al.* 2008). It is important to recognize that the consequences of one infection extending the length of another – in terms of the period of time host fitness may be affected and the parasite transmitted – may easily be as profound as those of simply increasing susceptibility. In this subsequent study, however, there was no evidence of the reverse interaction: *Bartonella* increasing susceptibility to *B. microti*. This runs counter to the Telfer *et al.* (2010) study and suggests that the effect of *Bartonella* on *B. microti* noted by the authors may be a statistical artefact arising from the extremely strong effects of *B. microti* on *Bartonella* (Sherlock *et al.* 2013).

This study also allowed interactions among different, co-infecting *Bartonella* species to be examined for the first time. Notably, voles that had previously been infected with *B. taylorii* were less likely to contract infections of either *B. grahamii* or *B. doshiae*. The suggestion that this positive interaction between the species may be the result of cross-immunity is supported by evidence from the analysis of an effective immune response to *Bartonella* infections more generally: voles previously infected with either *B. grahamii* or *B. taylorii* were less likely to re-contract the same infection. This in turn makes the more general point that patterns of co-infection, particularly in longitudinal data, can suggest or even support particular processes giving rise to them, but understanding co-infection is likely, ultimately, to require those processes to be examined directly. One important class of processes, those acting through the immune system, is examined next.

## IMMUNOLOGY

Traditionally, research into wildlife immunology has concentrated on broad definitions and single measurements of ‘immunocompetence’ (such as phytohaemagglutinin-induced swelling). However, as we have discussed in previous sections, host–pathogen interactions are dynamic and context dependent; ‘resistance’ is therefore unlikely to be accurately represented by a single, simplified immunological measure (Demas *et al.* 2011). Post-genomic technologies now allow us to define immune variability much more precisely in naturally occurring non-model organisms and move beyond this simplified view of immunocompetence. Wild rodents, in particular, represent an exciting model for this expanded ‘wild immunology’ as researchers can capitalize on the immunological and genetic resources developed for laboratory rodents. Thus, measurements of the expression of genes or gene products underpinning immunological traits may be linked to environmental causes and to life history consequences for the individual. In the Kielder field voles, where responses to infection have been the central interest, we have focused on variability in the immune system as the possible key to individual variation in the response to infection.

Our approach to measurement has made a break from traditional ecological immunology (Bradley and Jackson, 2008) by considering immune function explicitly from a perspective derived from studies of the laboratory mouse. Thus, the immune system is considered as a multi-faceted defensive apparatus with different arms that drive different types of immune responses. For example, this includes the different T-helper (Th) cell phenotypes: Th1 cells drive responses against intracellular microbes, Th2 cells those against macroparasites, Th17 cells drive responses against extracellular bacteria, and regulatory Th cells exert immunosuppressive responses (Reiner, 2007). These different arms of the immune system may trade-off with each other and with other life history components for resources and there may also be complex functional cross-talk (cross-regulation) within the immune system itself and between immune responses and other traits.

The broad aim of the Kielder immunology studies has been to analytically decompose immune system function through measurements of different effector arms and to link these measurements to environmental causes and life history responses. Developing appropriate measurement strategies is a central difficulty, though, in analysing the immune system in naturally occurring non-model organisms. In our initial studies we were hampered by the lack of species-specific antibody reagents and by a deficit of genomic information for *M. agrestis*. However, by *de novo* sequencing using traditional PCR methods, we were able to design real-time PCR expression assays for a panel of immunological genes reflecting different immunological pathways (Jackson *et al.* 2011). These measurements were used both on *in vivo* (peripheral blood) samples from repeat-sampled animals and in cultured splenocytes from destructively sampled animals. Culturing of splenocytes allowed stimulation of the cells with defined stimulants (e.g. mitogen, Toll-like receptor (TLR) agonists), in order to selectively stimulate immunological pathways and cell populations and measure latent (un-deployed) responsiveness (Jackson *et al.* 2011).

We combined these measurement approaches with interwoven longitudinal and cross-sectional sampling protocols in replicated habitats (see *Datasets*), for which detailed infection and biometric variables were recorded, in addition to immunological measurements. This hybrid study design exploited the respective strengths of the different types of sampling: on the one hand, destructive cross-sectional sampling allows a wider range of more precise immunological, biometric and infection measurements; on the other, longitudinal sampling allows stronger inference of cause and effect (cause typically preceding effect in time series data).

A preliminary proof-of-concept study based on a single season of cross-sectional data (2008–2009) from two field sites discovered significant non-periodic temporal trends in immune gene expression (Jackson *et al.* 2011). Expression of interferon regulatory factor 5 (*Irf5*), which is important in anti-viral and pro-inflammatory anti-microbial responses (Paun *et al.* 2008), declined across the study period. Some other genes, including transforming growth factor beta 1 (*Tgfb1*; a regulatory cytokine) and interleukin 2 (*Il2*), interferon gamma (*Ifng*) and T-box 21 (*Tbx21*/*Tbet*) (each involved in Th1 responses) showed expression that was higher in the winter of 2009 than the preceding winter. Both of these observations suggest systematic differences in environmental pressures on the immune system from year to year. Other variation (for example, expression of interleukin-1β [*Il1b*], involved in innate inflammatory responses) followed a pattern not inconsistent with a predictable annual/seasonal oscillation, although this would require corroboration in a longer time series. At the level of individuals we have found negative associations between the expression of pro-inflammatory mediators (*Ifng, Il1b*) and some individual condition indices (haematocrit and organ weight residuals on body length). This might, in part, reflect a cost of resistance mediated by inflammation or the adverse effect of immunogenic infections themselves. We also found significant variation between life history stages (especially between immature and mature males and between pregnant and other females).

In the first instance, this early analysis (which did not consider infection variables) has shown that the measurement approaches we adopted can identify systematic variation in the immune expression patterns of populations and individuals. It will be the aim of our future work to use longer observational time series (a full 2 years of cross-sectional and longitudinal immune expression, biometric and infection data are currently available) and experimental field manipulations to link this variation to its environmental drivers and to its consequences for co-infection patterns and life history traits in individuals. In doing so we will begin to better understand the immune system as a complex trait within an ecological context, but may also be able to use immune expression responses as biomarkers to track important ecological processes. Indeed, as our analyses have progressed to consider our full 2-year ‘immunodynamics’ dataset in the light of infection variables, a key finding has related to the immunological basis of disease tolerance to some infection types in wild field voles (Jackson *et al.* submitted). Disease tolerance is a defence mechanism whereby the host endures infection whilst minimizing the damage caused by the pathogen itself or by the host's own immune response (Ayres and Schneider, 2012; Medzhitov *et al.* 2012). This is distinct from disease resistance, where the host actively detects and eliminates pathogens, in that tolerance has no obvious effect on pathogen burden. Although the mechanisms of resistance are well understood, there is still relatively little known about the natural ability of animals to tolerate infection. Studies of natural populations are thus likely to provide a great many insights into this somewhat neglected defence mechanism (Turner and Paterson, 2013).

The results briefly described in this section have begun to identify important processes in host–parasite relationships that result from variation in immune function and were not immediately apparent from a simpler focus on just the parasites and the hosts themselves. In the future, continuing development of immunological methods for *M. agrestis* and the possibility to monitor the expression of many more genes using RNAseq, very high throughput Q-PCR and bioplexing are likely to further extend our understanding of the strategic role of the immune system within life history variation.

## GENETICS, SELECTION AND DISEASE SUSCEPTIBILITY

It is now well established that genetic diversity underlies a substantial component of the variation in susceptibility to infectious disease observed in natural populations. Just as they have for immunological studies, laboratory rodents have proved an invaluable resource in the discovery and functional annotation of genes involved in immunity to infection. However, although these animals are well-established functional genetic models, they differ from natural populations – including those of humans – in several important ways. First, laboratory rodents are generally inbred and therefore lack the genetic diversity of natural populations; second, genetic variation between laboratory strains is driven by selective breeding and deliberate mutations of the genome, rather than natural selection and genetic drift in the wild; third, laboratories provide homogeneous, comfortable and largely sterile environments with none of the pressures of the natural environment (for example, suboptimal nutrition, fluctuating climate, predation, competition etc.); and finally, laboratory infection experiments have tended to concentrate on single infections, whereas individuals in the natural environment are likely to experience multiple simultaneous or sequential infections by a taxonomically diverse set of parasites. Because of this lack of ecological validity, functional laboratory studies – while providing numerous mechanistic insights – offer few insights into the causes and consequences of natural genetic diversity, or the role of natural selection in the maintenance of variation in immune function and disease resistance. Wild rodents, in contrast, are related to laboratory rodents already well-established as genetic models and yet provide a much more realistic ecological model of human and other natural populations. They have therefore been put forward as a novel model to utilize and build on the genetic resources gained from their laboratory cousins, thus providing biomedically relevant yet ecologically valid insights into the genetic determinants of immunity and infectious disease susceptibility (Turner and Paterson, 2013).

In an attempt to broaden the immunogenetic research traditionally conducted on laboratory rodents to natural populations, Turner *et al.* (2011) used the longitudinal and cross-sectional system of Jackson *et al.* (2011) (see *Immunology* and *Datasets*) to examine the genetic diversity within a number of Kielder field vole immune genes.

Turner *et al.* concentrated primarily on cytokines, signalling molecules that facilitate communication between immune cells and which are crucial in the induction and polarization of immune responses. Despite the breadth of genetic research into cytokines in human and laboratory studies, there have thus far been relatively few studies on wildlife, where the overwhelming majority of immunogenetic studies have concentrated on the major histocompatibility complex (MHC) (Acevedo-Whitehouse and Cunningham, 2006).

The little previous research on the impact of cytokine genetic variation on parasite resistance in natural populations had concentrated on non-coding regions of a single gene, *Interferon gamma* (Coltman *et al.* 2001; Ezenwa *et al.* 2010). In their study, Turner *et al.* (2011) examined genetic polymorphism within protein-coding regions of a number of immune loci, and its association with individual variation in both immune responses and susceptibility to endemic pathogens. The authors utilized multiple regression methods to first control for confounding non-genetic factors, many of which were identified in the immunodynamics study of Jackson *et al.* (2011). Turner *et al.* demonstrated strong associations between genetic polymorphism within three cytokines (*Interleukin 1 beta* [*Il1b*], *Il2* and *Il12b*) and individual variation in immune responses, as measured through expression levels of multiple immune genes. Following this, the authors hypothesized that if genetic variation at cytokine loci affects immune responses, it would likely also impact upon susceptibility to pathogens. To test this, Turner *et al.* again first controlled for possible confounding factors, and subsequently found that the same three genes associated with variation in immune responses – *Il1b, Il2* and *Il12b* – were also strongly associated with variation in susceptibility to a number of endemic micro- and macroparasites. The magnitude of the genetic effects on both immune responses and pathogen resistance were of comparable size to non-genetic factors that are commonly acknowledged as important in natural studies of infection, such as age and sex. Importantly, given the importance of simultaneous infections in the Kielder voles (see *Co-infection*), all genetic effects remained after addition of co-infecting parasites to the models as explanatory variables. Moreover, the fact that these genes were associated with resistance to a taxonomically diverse range of natural pathogens (bacteria, protozoa, helminths and arthropod ectoparasites) demonstrates the value of examining such genetic relationships in the wild, in contrast to laboratory studies that typically focus on single experimental infections (Turner *et al.* 2011). For example, pleiotropic and apparently antagonistic effects of genetic variation were noted, with genetic variants simultaneously associated with an increased likelihood of infection with one parasite and a decreased chance of infection by another, a trade-off which has also been noted among different laboratory mouse genotypes (Pedersen and Babayan, 2011). This suggests that the advantage conferred on the host by a ‘protective’ genotype against one pathogen depends greatly on the context of the local pathogen community.

**Fig. 5.**
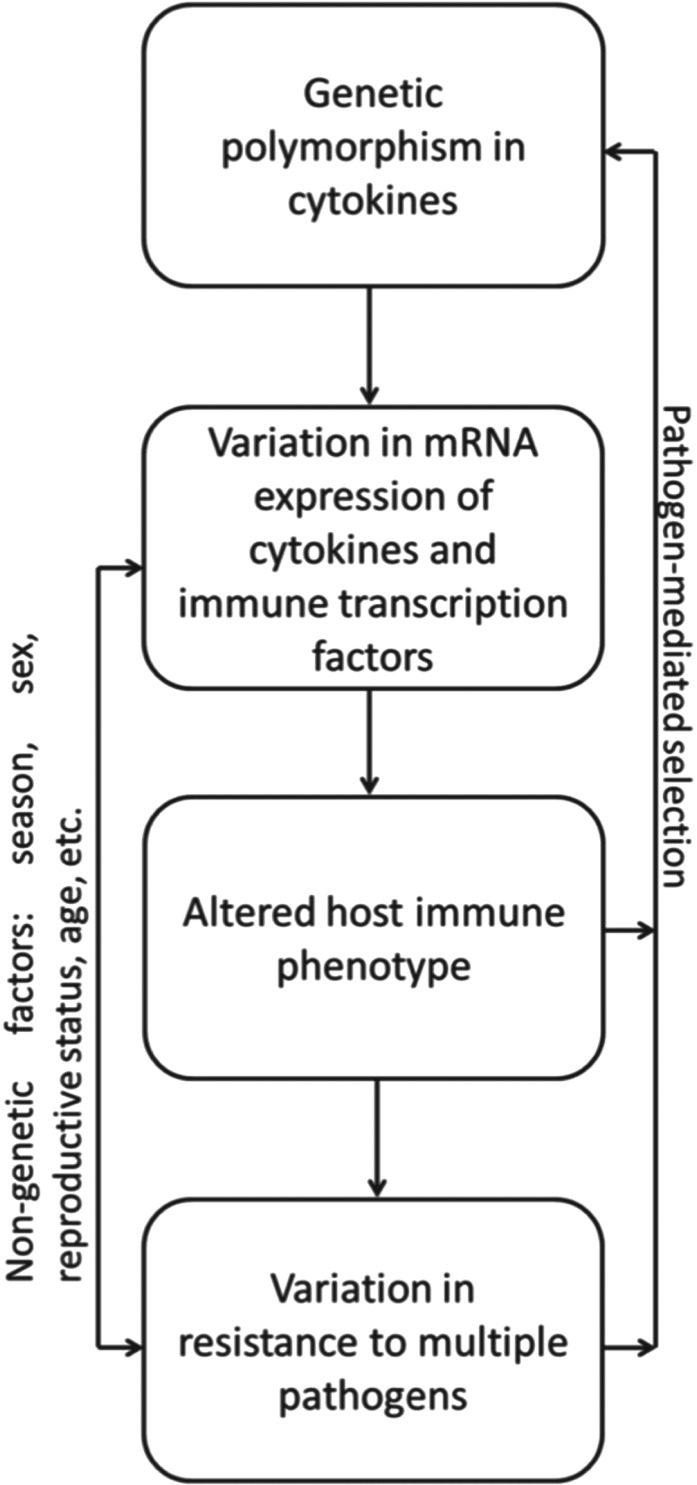
Causes and consequences of immunogenetic variation in Kielder voles. Polymorphism within cytokine genes – interacting with non-genetic factors – has a discernible effect on the transcription of immune genes and thus on host immune phenotype. Phenotypic variation in immune responses leads to variation among individuals in resistance to a taxonomically diverse range of endemic pathogens, the selective pressures of which drive the maintenance of cytokine genetic diversity. From Turner and Paterson (2013).

Understanding how the considerable heterogeneity among individuals in immune function and disease susceptibility is generated and maintained is a central question in evolutionary biology. In a complementary study to that above, Turner *et al.* (2012) therefore examined the role of natural selection in shaping diversity within field vole immune genes. Using a range of population genetic techniques they discovered signatures of natural selection acting on several cytokine and TLR genes. Of particular note was that high levels of genetic diversity observed within *Il1b* and *Il2* genes, both of which were strongly associated with variation in immune function and pathogen susceptibility, appear to have been maintained via balancing selection (a term encompassing any type of natural selection which acts to maintain genetic variation). There are several forms of balancing selection (including overdominance and frequency-dependent selection) and disentangling which mechanism(s) are the most important is challenging (Spurgin and Richardson, 2010). However, as pathogen abundances vary spatiotemporally in Kielder Forest (see for example Cavanagh *et al.* 2004; Telfer *et al.* 2007*a*, *b*), there are likely to be multiple pathogen-specific – and often competing – selection pressures acting concurrently. As such, fluctuating selection for different alleles at different points in space and/or time is the most likely mechanism driving the maintenance of polymorphism at these loci, particularly given the antagonistic and pleiotropic effects of genetic variation observed (Turner *et al.* 2011, 2012). Integration of the findings of the two studies by Turner *et al.* therefore provides robust, corroborative evidence that genetic diversity at cytokine loci has a discernible effect on susceptibility to a number of infectious diseases, via cytokine-mediated modulation of host immune phenotypes. In turn, as has been commonly reported for genes within the MHC (Piertney and Oliver, 2006; Spurgin and Richardson, 2010), cytokine genetic diversity is then maintained through the action of pathogen-mediated balancing selection (Fig. 5).

## CONCLUSIONS

Studies of infectious diseases in wild rodent populations have traditionally been driven by perhaps two major motivations (Begon, 2003). First, a fundamental desire to understand the ecological importance of the interactions between hosts and their parasites and, second, a more applied goal of understanding the dynamics of rodent reservoirs and their pathogens in order to practice disease control. In the first case, the work at Kielder, and indeed the work on bank voles and wood mice that spawned it, has made important connections between theoretical and conceptual ideals and the realities of the living world. Traditional dogmas and real data have been brought into contact with one another with regard, for example, to transmission functions, cross-species transmission, apparent competition, co-infection and the place of parasites in the web of a host's interactions more generally. In the second case, while many of the focal parasites have been zoonotic in principle, their public health importance in the UK has been limited. Nonetheless, techniques and approaches developed at Kielder have proved their value, and their transferability, when applied elsewhere, most notably perhaps to the plague–gerbil system in Central Asia (e.g. Davis *et al.* 2004, 2008).

With the advent of genomic technologies and the continued rise of ecological (‘wild’) immunology (Sheldon and Verhulst, 1996; Schulenburg *et al.* 2009; Martin *et al.* 2011; Pedersen and Babayan, 2011), a further motivation has now emerged: to expand traditional genetic and immunological research beyond laboratory models and into the natural world. There is an increasing recognition that fields that developed in relative isolation, such as molecular biology, ecology, immunology, epidemiology and evolutionary biology are not as distinct as they once appeared (Schmid-Hempel, 2011). Adopting an integrative approach to research, utilizing knowledge and resources from several fields, presents an excellent opportunity to bridge the gap between laboratory studies and the natural world and to better understand the nature of host–parasite interactions on many levels. We hope that the long-term, multidisciplinary research on the Kielder field voles and other natural systems will continue to provide fresh insights relevant not only to evolutionary biology and ecology, but also to conservation biology and biomedical science.
